# Surveillance for *Candida auris* — United States, 2022–2024

**DOI:** 10.15585/mmwr.ss7504a1

**Published:** 2026-07-02

**Authors:** Jeremy A. W. Gold, Anna D. Baker, Kaitlin Benedict, Kaitlin Forsberg, Jessica E. Laury, Ria R. Ghai, Danielle A. Rankin, Maroya Spalding Walters, Shawn R. Lockhart, Sophie Jones, Meghan Lyman, Candida auris surveillance team members

**Affiliations:** ^1^Division of Foodborne, Waterborne, and Environmental Diseases, National Center for Emerging and Zoonotic Infectious Diseases, CDC; ^2^Division of Healthcare Quality Promotion, National Center for Emerging and Zoonotic Infectious Diseases, CDC

## Abstract

**Problem/Condition:**

*Candida auris* is an emerging yeast that is frequently resistant to antifungal drugs. *C.* *auris* can cause invasive infections associated with high mortality and can colonize patients asymptomatically, which facilitates transmission in health care settings. Since it was first reported in the United States in 2016, *C. auris* has been identified in multiple states, with increasing numbers of cases reported annually. Monitoring national trends in cases identified through clinical testing and screening for colonization is critical to guide infection prevention and control efforts.

**Period Covered:**

2022–2024.

**Description of System:**

State and jurisdictional health departments voluntarily report clinical and screening *C. auris* cases to CDC using standardized case definitions of the Council of State and Territorial Epidemiologists. Clinical cases are defined as detection of *C. auris* from specimens collected for diagnostic purposes; screening cases are defined as detection from colonization screening swabs. Cases were reported to CDC through the Research Electronic Data Capture (REDCap) or Data Collation and Integration for Public Health Event Response (DCIPHER) platforms. Data included patient age and sex, case type, specimen type (for clinical cases), health care facility type, Antimicrobial Resistance Laboratory Network geographic region, and specimen collection date. Analyses were descriptive and limited to cases with specimens collected during 2022–2024.

**Results:**

During 2022–2024, a total of 13,507 clinical *C. auris* cases were reported to CDC, increasing from 2,882 in 2022 to 4,428 in 2023 and 6,197 in 2024, with smaller annual percentage increases over time (53.7% from 2022 to 2023 and 39.9% from 2023 to 2024). Most clinical cases occurred among adults aged ≥45 years (87.8%) and among males (61.0%). The most common specimen types among all clinical cases were urine (31.5%) and blood (30.2%); by year, the proportion of blood as the specimen type was 34.4% in 2022, 30.2% in 2023, and 25.6% in 2024. Most clinical cases were identified through specimens collected in acute care hospitals (76.6%) and long-term acute care hospitals (17.8%).

During the same period, a total of 27,853 screening cases were reported to CDC, increasing from 6,226 in 2022 to 9,195 in 2023 and 12,432 in 2024. Screening cases most frequently occurred among adults aged ≥45 years (90.0%) and males (57.9%). Among cases with known facility type, the proportion of specimens collected in acute care hospitals increased from 24.7% in 2022 to 50.7% in 2024, whereas the proportion of specimens collected in long-term acute care hospitals decreased from 56.1% to 35.7% during the same period.

**Interpretation:**

The number of clinical and screening *C. auris* cases reported to CDC increased during 2022–2024, indicating ongoing transmission in U.S. health care settings. Although annual percentage increases in clinical cases declined over time, absolute case counts reported to CDC continued to rise. The increasing proportion of screening cases with specimens collected in acute care hospitals might reflect increased use of screening in acute care hospitals, including screening at admission.

**Public Health Action:**

Because of increases in the number of reported *C.* *auris* cases, sustained infection prevention and control efforts in health care facilities, including adherence to transmission-based precautions, environmental disinfection with agents effective against *C. auris*, and communication of *C. auris* status during patient transfers remain essential to preventing clinical infections and colonization. Because this pathogen is frequently resistant to antifungal drugs, continued investment in laboratory capacity and surveillance, including antifungal susceptibility testing and screening of patients at high risk for *C. auris* infection, can support timely detection and guide prevention strategies. Ongoing public health coordination at federal, state, and local levels is critical to limit further spread and to address emerging antifungal drug resistance.

## Introduction

*Candida auris* is an emerging, frequently drug-resistant yeast that can cause invasive infections with high mortality rates (*1*–*4*). It can colonize patients asymptomatically and persist on surfaces, facilitating rapid spread in health care settings, especially in acute care hospitals (ACHs), long-term acute care hospitals (LTACHs), and skilled nursing facilities (SNFs) equipped with a ventilator (*5*–*7*). Symptoms of *C. auris* infection are nonspecific and depend on the affected body site and infection severity, ranging from superficial skin infections to invasive forms such as bloodstream infections (i.e., candidemia) (*7*). Because most *C. auris* isolates are resistant to the azole antifungal drug fluconazole, a common treatment for candidal infections, echinocandin antifungals are first-line treatments (*8*). However, echinocandin-resistant and multidrug-resistant strains (resistant to azoles, echinocandins, and polyenes) have been detected in the United States; although echinocandin resistance remains uncommon, these strains can limit available treatment options (*9*–*11*).

Risk factors for *C. auris* colonization (i.e., presence on skin or mucosal surfaces without causing infection) and for invasive infection include frequent or prolonged stays in health care facilities, complex medical care involving invasive medical devices (e.g., mechanical ventilation and central venous catheters), and recent antimicrobial use (*12*). Screening for *C. auris* colonization in patients at high risk is a critical component of strategies to prevent spread in health care facilities because identification of colonized patients enables facilities to implement recommended infection prevention and control measures (*13*). Deciding which patients to screen might be based on multiple factors, including local *C. auris* epidemiology, epidemiologic linkages to other cases (e.g., those on the same unit), specific patient risk factors, and the purpose of screening (*14*). Screening can be performed using a broad approach by conducting a point prevalence survey (e.g., screening all patients or residents of a unit or facility) or a more targeted approach (e.g., screening certain patients on the basis of risk factors, epidemiologic links, or clinical characteristics) (*14*).

The first* C. auris* cases were reported in the United States in 2016 (*15*). Early cases were usually associated with health care exposure abroad, whereas most of the recent cases were acquired in U.S. health care settings (*1*,*16*). The number of reported clinical *C. auris* cases increased from approximately 50 in 2016 to approximately 1,500 in 2021; the largest percentage increase (95%) occurred from 2020 to 2021 (*1*,*16*). In certain states and jurisdictions, *C. auris* has become endemic, leading to sustained high rates of transmission and increasing prevalence across health care facilities. Meanwhile, other states and jurisdictions are just beginning to report their first cases (*16*).

A comprehensive understanding of the epidemiology of *C. auris* in the United States is critical to guide public health response efforts. This report summarizes national *C. auris* case report data for clinical and screening cases identified during 2022–2024. Public health officials and policymakers can use these findings to strengthen surveillance and public health responses, guide resource allocation, and focus initiatives aimed at enhancing patient safety in health care facilities.

## Methods

### Surveillance Case Definitions

Consistent with the Council of State and Territorial Epidemiologists (CSTE) 2023 *C. auris* case definitions (*17*), a clinical case was defined as the detection of *C. auris* from a patient specimen tested to determine the cause of a suspected infection. The specimen could be taken from blood, which typically indicates an invasive infection, or from other diagnostic specimen types (e.g., urine) that might represent an infection or colonization detected during clinical testing. A screening case was defined as the detection of *C. auris* from a swab, most often a composite 1) axilla and groin or 2) nares, axilla, and groin swab collected to test for colonization. A patient could be classified as having up to two cases: one screening case followed by one clinical case; analyses were conducted at the case level, and multiple positive specimens within a single case type were not counted as separate cases.

### Data Sources

Reporting requirements for *C. auris* vary by state and jurisdiction. State and jurisdictional health departments can voluntarily report clinical and screening *C. auris* cases to CDC. *C. auris* cases from states and jurisdictions were transmitted to CDC using either the secure Research Electronic Data Capture (REDCap) database (*18*,*19*) or Data Collation and Integration for Public Health Event Response (DCIPHER), a cloud-based CDC data platform. The data collected included information on patient age, sex, case type (i.e., clinical or screening), specimen type for clinical cases (i.e., blood, wound, urine, respiratory, or other), date of specimen collection, facility type, and Antimicrobial Resistance Laboratory Network (AR Laboratory Network) region of the facility where the specimen was collected. The AR Laboratory Network regions are *Central* (Arkansas, Iowa, Kansas, Minnesota, Missouri, Nebraska, North Dakota, Oklahoma, and South Dakota); *Mid-Atlantic* (Delaware, District of Columbia, Maryland, North Carolina, Pennsylvania, South Carolina, Virginia, and West Virginia); *Midwest* (Illinois, Indiana, Kentucky, Michigan, Ohio, and Wisconsin); *Mountain* (Arizona, Colorado, Idaho, Montana, New Mexico, Texas, Utah, and Wyoming); *Northeast* (Connecticut, Maine, Massachusetts, New Hampshire, New Jersey, New York, Rhode Island, and Vermont); *Southeast* (Alabama, Florida, Georgia, Louisiana, Mississippi, Puerto Rico, and Tennessee); and *West* (Alaska, California, Guam, Hawaii, Nevada, Oregon, and Washington).

In the United States, *C. auris* clinical cases have been nationally notifiable since 2019, and *C. auris* screening cases have been nationally notifiable since 2023. Nationally notifiable conditions are typically reported to CDC through the National Notifiable Diseases Surveillance System (NNDSS). However, data analyzed for this report were case-level data from CDC’s case-based surveillance system, which includes variables not available in NNDSS (e.g., facility type). Data included in these analyses were current as of February 20, 2026 (the date on which the analytic dataset for this report was finalized). CDC web-based surveillance summaries might display different case totals because of ongoing case reporting, data updates, and reconciliation.

### Analyses

Total *C. auris* case counts for specimens collected during 2022–2024 and annual counts by case type, age group, sex, AR Laboratory Network region, specimen type for clinical cases (i.e., blood, wound, urine, respiratory, or other), and facility type (i.e., LTACH, ACH, SNF, ventilator-equipped SNF, or other) at the time of specimen collection were examined. For each patient, only the first specimen (by date collected) per case type (i.e., screening or clinical) was included in analyses. *Candida* isolates from blood are usually identified to the species level, whereas species identification for nonblood isolates is dependent on local laboratory practices (e.g., implementation of enhanced surveillance to identify all yeast from urine); therefore, year-specific analyses of specimen type focused on blood specimens.

Analyses for this report were descriptive; statistical testing was not performed because denominator data were unavailable and testing and screening practices varied over time and across jurisdictions. The primary measure for this analysis was the number of reported *C. auris* cases by year of specimen collection. These counts are influenced by multiple factors, including changes in testing capacity, screening practices, and reporting across jurisdictions. Therefore, findings should be interpreted as descriptions of trends in reported cases within the surveillance system and not representative of population-based incidence. This activity was reviewed by CDC, deemed not research, and conducted consistent with applicable federal law and CDC policy.*

## Results

### Clinical *C. auris* Cases

A total of 13,507 clinical *C. auris* cases from specimens collected during 2022–2024 were reported to CDC (Table 1). The number of clinical *C. auris* cases reported to CDC increased from 2,882 in 2022 to 4,428 in 2023 to 6,197 in 2024. Overall, most clinical cases occurred among adults aged 45–64 years (32.2%) or 65–74 years (29.5%); 19.6% of cases occurred among those aged 75–84 years, 12.0% among those aged 18–44 years, 6.5% among adults aged ≥85 years, and 0.2% among those aged 0–17 years. Most cases were among males (61.0%). The most common specimen type was urine (31.5%), followed by blood (30.2%), wound (16.7%), and respiratory (13.1%). The majority of specimens were collected from the West AR Laboratory Network region (28.5%), the Midwest region (21.3%), or the Southeast region (20.2%). The most common facility type for specimen collection was ACH (76.6%), followed by LTACH (17.8%) and SNF (1.7%). Overall, data completeness varied across variables; the highest proportion of missing data was for facility type (missing for 13.1% of clinical cases and 12.5% of screening cases).

**TABLE 1:** Demographic characteristics of persons with reported *Candida auris*, number of clinical cases, and facility type in which specimen was collected — United States, 2022–2024*

Characteristic	Clinical cases
	No. (%)
**Year of specimen collection**	**13,507 (100)**
2022	2,882 (21.3)
2023	4,428 (32.8)
2024	6,197 (45.9)
**Age group, yrs**	**12,058 (100)**
0–17	21 (0.2)
18–44	1,447 (12.0)
45–64	3,886 (32.2)
65–74	3,557 (29.5)
75–84	2,365 (19.6)
≥85	782 (6.5)
**Sex **	**12,003 (100)**
Female	4,683 (39.0)
Male	7,320 (61.0)
**Specimen type among clinical cases**	**12,958 (100)**
Blood^†^	3,914 (30.2)
Respiratory	1,693 (13.1)
Urine	4,086 (31.5)
Wound	2,168 (16.7)
Other	1,097 (8.5)
**Antimicrobial Resistance Laboratory Network region^§^**	**13,507 (100)**
West	3,845 (28.5)
Midwest	2,875 (21.3)
Southeast	2,733 (20.2)
Northeast	1,571 (11.6)
Mountain	1,448 (10.7)
Mid-Atlantic	900 (6.7)
Central	135 (1.0)
**Facility type where specimen was collected^¶^**	**11,740 (100)**
Acute care hospital	8,994 (76.6)
Long-term acute care hospital	2,084 (17.8)
Skilled nursing facility, not ventilator equipped	194 (1.7)
Skilled nursing facility, ventilator equipped	91 (0.8)
Other	377 (3.2)

* A clinical case was defined as the detection of *Candida auris* from a patient specimen tested to determine the cause of an infection rather than to detect colonization. Category totals might not sum to the overall number of clinical cases because of missing data. Percentages were calculated using the number of cases with complete information for each variable as the denominator. Antimicrobial Resistance Laboratory Network region had no missing data in any year.

^†^ The following number of blood specimens were collected from clinical cases, by year: in 2022, a total of 991 (34.4%); in 2023, a total of 1,337 (30.2%); and in 2024, a total of 1,586 (25.6%).

^§^ Antimicrobial Resistance Laboratory Network region of the facility where the specimen was collected. Jurisdictions within each network region are as follows: *Central* (Arkansas, Iowa, Kansas, Minnesota, Missouri, Nebraska, North Dakota, Oklahoma, and South Dakota); *Mid-Atlantic* (Delaware, District of Columbia, Maryland, North Carolina, Pennsylvania, South Carolina, Virginia, and West Virginia); *Midwest* (Illinois, Indiana, Kentucky, Michigan, Ohio, and Wisconsin); *Mountain* (Arizona, Colorado, Idaho, Montana, New Mexico, Texas, Utah, and Wyoming); *Northeast* (Connecticut, Maine, Massachusetts, New Hampshire, New Jersey, New York, Rhode Island, and Vermont); *Southeast* (Alabama, Florida, Georgia, Louisiana, Mississippi, Puerto Rico, and Tennessee); and *West* (Alaska, California, Guam, Hawaii, Nevada, Oregon, and Washington). Antimicrobial Resistance Laboratory Network | CDC

^¶^ Does not necessarily indicate where acquisition or transmission of *C. auris* occurred.

Of the specimen types among clinical cases, blood accounted for 34.4% (991 of 2,882) in 2022, 30.2% (1,337 of 4,428) in 2023, and 25.6% (1,586 of 6,197) in 2024 (Tables 1 and 2). Among clinical cases with blood specimens, age, sex, and facility type distributions were similar across years. Regional percentage distributions were also comparable across years, but changes in percentages were observed from 2022 to 2024, including in the Northeast (21.8% of cases in 2022 to 13.7% in 2024).

**TABLE 2:** Demographic characteristics of persons with reported *Candida auris*, number of clinical cases involving blood specimens, and facility type in which specimen was collected, by year — United States, 2022–2024*

Characteristic	2022	2023	2024	Total
	No. (%)	No. (%)	No. (%)	No. (%)
**Age group, yrs **	**782 (100)**	**1,143 (100)**	**1,419 (100)**	**3,344 (100)**
0–17	2 (0.3)	2 (0.2)	2 (0.1)	**6 (0.2)**
18–44	99 (12.7)	151 (13.2)	176 (12.4)	**426 (12.7)**
45–64	265 (33.9)	383 (33.5)	489 (34.5)	**1,137 (34.0)**
65–74	229 (29.3)	333 (29.1)	418 (29.5)	**980 (29.3)**
75–84	139 (17.8)	226 (19.8)	265 (18.7)	**630 (18.8)**
≥85	48 (6.1)	48 (4.2)	69 (4.9)	**165 (4.9)**
**Sex **	**777 (100)**	**1,141 (100)**	**1,413 (100)**	**3,331 (100)**
Female	353 (45.4)	499 (43.7)	649 (45.9)	**1,501 (45.1)**
Male	424 (54.6)	642 (56.3)	764 (54.1)	**1,830 (54.9)**
**Antimicrobial Resistance Laboratory Network region^†^**	**991 (100)**	**1,337 (100)**	**1,586 (100)**	**3,914 (100)**
West	269 (27.1)	341 (25.5)	386 (24.3)	**996 (25.4)**
Southeast	191 (19.3)	268 (20.0)	350 (22.1)	**809 (20.7)**
Midwest	188 (19.0)	254 (19.0)	282 (17.8)	**724 (18.5)**
Northeast	216 (21.8)	219 (16.4)	218 (13.7)	**653 (16.7)**
Mountain	60 (6.1)	166 (12.4)	191 (12.0)	**417 (10.7)**
Mid-Atlantic	67 (6.8)	80 (6.0)	134 (8.4)	**281 (7.2)**
Central	0 (—)	9 (0.7)	25 (1.6)	**34 (0.9)**
**Facility type where specimen was collected^§^**	**839 (100)**	**1,167 (100)**	**1,391 (100)**	**3,397 (100)**
Acute care hospital	644 (76.8)	933 (79.9)	1,072 (77.1)	**2,649 (78.0)**
Long-term acute care hospital	170 (20.3)	212 (18.2)	278 (20.0)	**660 (19.4)**
Skilled nursing facility, not ventilator equipped	7 (0.8)	5 (0.4)	12 (0.9)	**24 (0.7)**
Skilled nursing facility, ventilator equipped	10 (1.2)	9 (0.8)	11 (0.8)	**30 (0.9)**
Other	8 (1.0)	8 (0.7)	18 (1.3)	**34 (1.0)**

* A clinical case was defined as the detection of *Candida auris* from a patient specimen tested to determine the cause of an infection rather than to detect colonization. Category totals might not sum to the total values because of missing data. Percentages were calculated using the number of cases with complete information for each variable as the denominator. Antimicrobial Resistance Laboratory Network region had no missing data in any year.

^†^ Antimicrobial Resistance Laboratory Network region of the facility where the specimen was collected. Jurisdictions within each network region are as follows: *Central* (Arkansas, Iowa, Kansas, Minnesota, Missouri, Nebraska, North Dakota, Oklahoma, and South Dakota); *Mid-Atlantic* (Delaware, District of Columbia, Maryland, North Carolina, Pennsylvania, South Carolina, Virginia, and West Virginia); *Midwest* (Illinois, Indiana, Kentucky, Michigan, Ohio, and Wisconsin); *Mountain* (Arizona, Colorado, Idaho, Montana, New Mexico, Texas, Utah, and Wyoming); *Northeast* (Connecticut, Maine, Massachusetts, New Hampshire, New Jersey, New York, Rhode Island, and Vermont); *Southeast* (Alabama, Florida, Georgia, Louisiana, Mississippi, Puerto Rico, and Tennessee); and *West* (Alaska, California, Guam, Hawaii, Nevada, Oregon, and Washington). Antimicrobial Resistance Laboratory Network | CDC

^§^ Does not necessarily indicate where acquisition or transmission of *C. auris* occurred.

### Screening *C. auris* Cases

A total of 27,853 screening cases with specimens collected during 2022–2024 were reported to CDC (Table 3). The number of screening cases reported to CDC increased from 6,226 in 2022 to 9,195 in 2023 to 12,432 in 2024. Overall, most screening cases occurred among adults aged 45–64 years (30.6%) or 65–74 years (29.5%); 21.3% of cases occurred among adults aged 75–84 years, 10.0% among those aged 18–44 years, 8.5% among those aged ≥85 years, and <0.1% among those aged 0–17 years. Most (57.9%) screening cases were among males, and the largest proportion of screening specimens were collected from the West AR Laboratory Network region (36.0%), followed by the Southeast (18.8%) and Midwest regions (18.8%). The most common facility type for specimen collection was LTACH (42.1%), followed by ACH (41.8%) and ventilator-equipped SNF (10.3%).

**TABLE 3:** Demographic characteristics of persons with reported *Candida auris*, number of screening cases, and facility type in which specimen was collected, by year — United States, 2022–2024*

Characteristic	2022	2023	2024	Total
	No. (%)	No. (%)	No. (%)	No. (%)
**Age group, yrs **	**5,390 (100)**	**8,569 (100)**	**11,821 (100)**	**25,780 (100)**
0–17	2 (<0.1)	3 (<0.1)	4 (<0.1)	**9 (<0.1)**
18–44	497 (9.2)	926 (10.8)	1,156 (9.8)	**2,579 (10.0)**
45–64	1,688 (31.3)	2,644 (30.9)	3,568 (30.2)	**7,900 (30.6)**
65–74	1,617 (30.0)	2,480 (28.9)	3,500 (29.6)	**7,597 (29.5)**
75–84	1,110 (20.6)	1,823 (21.3)	2,567 (21.7)	**5,500 (21.3)**
≥85	476 (8.8)	693 (8.1)	1,026 (8.7)	**2,195 (8.5)**
**Sex **	**5,268 (100)**	**8,445 (100)**	**11,681 (100)**	**25,394 (100)**
Female	2,277 (43.2)	3,540 (41.9)	4,884 (41.8)	**10,701 (42.1)**
Male	2,991 (56.8)	4,905 (58.1)	6,797 (58.2)	**14,693 (57.9)**
**Antimicrobial Resistance Laboratory Network region^†^**	**6,226 (100)**	**9,195 (100)**	**12,432 (100)**	**27,853 (100)**
West	2,242 (36.0)	3,208 (34.9)	4,575 (36.8)	**10,025 (36.0)**
Southeast	1,167 (18.7)	2,018 (21.9)	2,054 (16.5)	**5,239 (18.8)**
Midwest	1,111 (17.8)	1,676 (18.2)	2,444 (19.7)	**5,231 (18.8)**
Northeast	824 (13.2)	952 (10.4)	1,274 (10.2)	**3,050 (11.0)**
Mountain	406 (6.5)	774 (8.4)	1,099 (8.8)	**2,279 (8.2)**
Mid-Atlantic	473 (7.6)	522 (5.7)	734 (5.9)	**1,729 (6.2)**
Central	3 (<0.1)	45 (0.5)	252 (2.0)	**300 (1.1)**
**Facility type where specimen was collected^§^**	**5,438 (100)**	**7,894 (100)**	**11,041 (100)**	**24,373 (100)**
Acute care hospital	1,345 (24.7)	3,235 (41.0)	5,598 (50.7)	**10,178 (41.8)**
Long-term acute care hospital	3,048 (56.1)	3,278 (41.5)	3,937 (35.7)	**10,263 (42.1)**
Skilled nursing facility, not ventilator equipped	229 (4.2)	343 (4.3)	466 (4.2)	**1,038 (4.3)**
Skilled nursing facility, ventilator equipped	722 (13.3)	914 (11.6)	877 (7.9)	**2,513 (10.3)**
Other	94 (1.7)	124 (1.6)	163 (1.5)	**381 (1.6)**

* A screening case was defined as the detection of *Candida auris* from a swab collected to test for colonization in a patient. A patient could be classified as having up to two cases: one screening case followed by one clinical case. Category totals might not sum to the overall number of screening cases because of missing data. Percentages were calculated using the number of cases with complete information for each variable as the denominator. Antimicrobial Resistance Laboratory Network region had no missing data in any year.

^†^ Antimicrobial Resistance Laboratory Network region of the facility where the specimen was collected. Jurisdictions within each network region are as follows: *Central* (Arkansas, Iowa, Kansas, Minnesota, Missouri, Nebraska, North Dakota, Oklahoma, and South Dakota); *Mid-Atlantic* (Delaware, District of Columbia, Maryland, North Carolina, Pennsylvania, South Carolina, Virginia, and West Virginia); *Midwest* (Illinois, Indiana, Kentucky, Michigan, Ohio, and Wisconsin); *Mountain* (Arizona, Colorado, Idaho, Montana, New Mexico, Texas, Utah, and Wyoming); *Northeast* (Connecticut, Maine, Massachusetts, New Hampshire, New Jersey, New York, Rhode Island, and Vermont); *Southeast* (Alabama, Florida, Georgia, Louisiana, Mississippi, Puerto Rico, and Tennessee); and *West* (Alaska, California, Guam, Hawaii, Nevada, Oregon, and Washington). Antimicrobial Resistance Laboratory Network | CDC

^§^ Does not necessarily indicate where acquisition or transmission of *C. auris* occurred.

From 2022 to 2024, the distribution patterns for age, sex, and region were comparable. Among screening cases with known facility type (n = 24,373 [87.5%]; facility type was unknown for 12.5% of cases), the percentage of cases from ACHs changed from 24.7% (n = 1,345 of 5,438) in 2022 to 41.0% (n = 3,235 of 7,894) in 2023 to 50.7% (n = 5,598 of 11,041) in 2024, whereas the percentage from LTACHs changed from 56.1% (n = 3,048 of 5,438) in 2022 to 41.5% (n = 3,278 of 7,894) in 2023 to 35.7% (n = 3,937 of 11,041) in 2024.

## Discussion

This analysis of U.S. national surveillance data revealed that 2,882 clinical cases of *C. auris* were reported to CDC in 2022, representing an approximate 95.9% increase compared with 2021 (*1*). Case counts continued to increase in 2023, with 4,428 cases reported, and in 2024, with 6,197 cases, but the annual changes were smaller over time: 95.9% (2021 to 2022), 53.7% (2022 to 2023), and 39.9% (2023 to 2024). The pronounced increase in clinical case counts from 2021 to 2022 might have been influenced by multiple factors, including strains on health care systems caused by the COVID-19 pandemic, which resulted in shortages of supplies and personnel, as well as overcrowding (*20*). In addition, more patients might have been at risk for *C. auris* colonization and infection during this period because of the widespread use of broad-spectrum antibiotics to treat secondary bacterial infections associated with COVID-19 and the increased population of critically ill patients receiving mechanical ventilation and having prolonged stays at health care facilities (*21*). The smaller percentage increases observed after 2022 might reflect, in part, a renewed focus on standard protocols aimed at mitigating the spread of *C. auris* and other health care–associated infections, particularly after the resolution of personal protective equipment shortages and staffing challenges experienced during the earlier phases of the COVID-19 pandemic. Improved testing capacity might also have contributed to greater detection of clinical *C. auris* cases over time as laboratories have increasingly adopted species-level identification or updated laboratory equipment (e.g., testing libraries) to properly identify *C. auris*. Increased screening, particularly in ACHs, and changes in testing and reporting practices over time might also have contributed (*22*,*23*). However, the relative contribution of these factors to the observed increases cannot be quantified with the available data.

The continued increase in clinical *C. auris* case counts reported to CDC during 2022–2024 underscores the establishment and ongoing transmission of this pathogen. Clinical cases most frequently involved specimens collected from patients in ACHs (76.6%) and LTACHs (17.8%), although the data do not indicate where transmission might have occurred. This finding highlights the importance of strict adherence to infection prevention and control measures in health care facilities, which is bolstered by sustained support from state and local health departments, as well as federal agencies, through guidance, technical assistance, and site visits (*24*). CDC recommendations involve several core components of infection prevention and control in health care settings, including rigorous hand hygiene practices, the use of disinfectants effective against *C. auris* in facilities caring for patients with *C. auris*, and the implementation of transmission-based precautions, such as contact precautions in ACHs and LTACHs and either contact precautions or enhanced barrier precautions in nursing homes and skilled nursing facilities, depending on the setting and jurisdictional recommendations (*24*). CDC also recommends regular audits of infection control practices and measures to ensure effective communication among health care facilities of patient *C. auris* status during transfers (*24*). In general, facilities that are equipped to care for patients with other multidrug-resistant organisms or *Clostridioides difficile* can also effectively care for patients with *C. auris* (*24*).

Approximately one third of reported clinical cases (30.2%) were identified from blood specimens. Because isolating *Candida* from blood indicates an invasive infection, this result is concerning because of the pathogen’s frequent antifungal drug resistance and high mortality (30%–72%) (*25*–*27*). Trends in nonblood specimen types should be interpreted with caution because changes in laboratory practices, such as expanded species-level identification of yeast from nonsterile sources (e.g., urine), might influence their relative distribution over time. These findings underscore the importance of efforts to prevent health care–associated *Candida* bloodstream infections. Consistent with previous data, clinical cases predominantly occurred in adults aged ≥45 years, with most cases identified in the West (28.5%), Midwest (21.3%), and Southeast (20.2%) regions of the AR Laboratory Network (*1*). Clinical *C. auris* cases were more common among males (61.0%); the reasons for this pattern are unclear and were not evaluated in this surveillance analysis.

The number of screening *C. auris* cases reported to CDC increased from 6,226 in 2022 to 12,432 in 2024, and by 2024, screening cases were most frequently detected in ACHs. Initially, *C. auris* screening programs and containment efforts focused on LTACHs and ventilator-equipped SNFs, because these were identified as settings at high risk for *C. auris* colonization and outbreaks (*28*,*29*). This risk is largely because of their patient populations, which often include patients receiving mechanical ventilation, experiencing prolonged hospital stays, and with severe underlying conditions (*13*,*28*). The reason for the shift in screening case detection is uncertain; however, it might indicate increased use of screening in ACHs to identify colonized patients during admission, rather than reflecting actual transmission within ACHs (*23*). Consequently, ACHs might be identifying colonized patients at the time of admission through expanded in-house or admission screening protocols or policies, even when colonization acquisition occurred at another facility (*1*). Finally, screening cases became nationally notifiable in 2023, which might have improved completeness of reporting and contributed to increases observed after 2022.

Overall, because the number of *C. auris* screening cases is dependent on local screening practices, which vary among facilities and regions (*30*), determining the degree to which increased screening case counts reflect a true increase in disease prevalence rather than sampling bias is difficult. The increase in screening cases might be caused by heightened awareness and enhanced surveillance efforts as more institutions implement routine screening protocols. This increase might be linked to the adoption by public health agencies and health care facilities of CDC guidelines, which promote targeted and, when warranted, facilitywide screening for *C. auris*, with increased frequency among populations at high risk for infection on the basis of local epidemiology and transmission risk (*14*). In addition, advancements in diagnostic testing, particularly the increased use of polymerase chain reaction–based tests that are more rapid and sensitive than traditional culture-based methods, might also have contributed to increased screening case detection (*31*–*33*).

## Limitations

The findings in this report are subject to several limitations. First, *C. auris* case counts in this report might differ from those reported publicly by individual state and jurisdictional health departments or other surveillance platforms, which might limit direct comparisons. Differences across data sources might reflect variations in surveillance systems (e.g., REDCap and NNDSS), surveillance case definitions, inclusion criteria (e.g., individual surveillance cases, which might represent more than one case per patient; laboratory specimens; or unique patients), assignment on the basis of patient residence versus location of specimen collection, and use of report date versus specimen collection date. Second, although data in CDC’s case-based surveillance system are reported using standardized definitions (i.e., CSTE surveillance case definitions), which supports consistency across jurisdictions, these standardized data are not intended to replicate jurisdiction-specific public reporting. Continued efforts to align reporting platforms and required data elements across jurisdictions could enhance data completeness and standardization. Third, because clinical cases are defined on the basis of detection of *C. auris* from specimens collected for diagnostic purposes, rather than on the basis of clinical evidence of infection, certain specimen types, particularly urine and respiratory specimens, might represent colonization rather than true infection in the absence of detailed clinical information. Fourth, certain denominator data, including the number of patients tested or screened, the number of participating facilities, and facility- or jurisdiction-level census data, were unavailable; therefore, incidence and prevalence could not be estimated, and changes over time might reflect changes in ascertainment rather than true changes in disease frequency. Fifth, the prevalence data in this report are likely underestimates of the true prevalence of *C. auris* because colonized patients might not be screened, and certain clinical infections might not be detected because cultures are not obtained or isolates are not identified at the species level. Sixth, *C. auris* screening cases became nationally notifiable in 2023, which might have increased completeness of reporting in 2023 compared with 2022 and affected interpretation of temporal trends. Seventh, jurisdiction-level changes in reporting requirements, as well as changes in testing and screening practices over time, might have influenced case counts; therefore, described trends should be interpreted with caution and might not fully reflect true changes in incidence or prevalence. Finally, the surveillance system does not include information on negative test results, race, ethnicity, socioeconomic information, treatments, outcomes, or antifungal susceptibility testing, which would be valuable for guiding public health surveillance and treatment guidelines (*1*,*4*).

## Future Directions

Sustained investment in laboratory testing capacity for *C. auris* identification and antifungal drug susceptibility testing, such as through the AR Laboratory Network and by other laboratories, could facilitate rapid case detection and evaluation of emerging trends such as changes in antimicrobial resistance patterns. Expanding testing capacity in clinical and commercial laboratories might further improve timely detection by bringing testing closer to health care facilities. Together, these investments might support enhanced surveillance efforts, which might help refine prevention strategies by identifying the most effective screening approaches for preventing transmission and understanding modifiable factors associated with the progression from colonization to infection (*34*). Enhanced surveillance might also direct clinical guidance by providing information on treatment practices, patient outcomes, and antifungal drug susceptibility testing patterns and results (*10*,*25*). Research initiatives might focus on identifying effective decolonization or pathogen reduction strategies to reduce the risk for infection among colonized patients and to disrupt *C. auris* transmission. In addition, initiatives could focus on optimizing recommended antimicrobial use and developing novel antifungal treatment options to expand the range of therapies available against resistant strains (*35*–*37*).

## Conclusion

*C. auris* remains a critical public health concern in the United States and continues to be most frequently identified among middle-aged and older adults, particularly in specimens collected from patients in ACHs and LTACHs. From 2022 to 2024, the number of clinical *C. auris* cases reported to CDC increased approximately twofold, from 2,882 to 6,197. Although the annual percent increase in reported cases decreased during the study period, the continued rise in cases underscores ongoing transmission in health care settings and the importance of infection prevention and control efforts, with continued support from federal, state, and local public health partners to prevent further spread.

## Footnotes

* 45 C.F.R. part 46.102(l)(2), 21 C.F.R. part 56; 42 U.S.C. Sect. 241(d); 5 U.S.C. Sect. 552a; 44 U.S.C. Sect. 3501 et seq.
