# *Erratum*: Vol. 75, No. SS-4

**DOI:** 10.15585/mmwr.mm7527a3

**Published:** 2026-07-16

The report “Surveillance for *Candida auris* — United States, 2022–2024” contained an error.

On page 6, the *Candida auris* surveillance team members listed for the New York State Department of Health should have read, “**Coralie Bucher, Warangkana Sangchan, Jeffrey Wu,**”.

